# Recent insights into the molecular mechanisms of simultaneous fatty acid oxidation and synthesis in brown adipocytes

**DOI:** 10.3389/fendo.2023.1106544

**Published:** 2023-02-21

**Authors:** Ji Suk Chang

**Affiliations:** Gene Regulation and Metabolism Laboratory, Pennington Biomedical Research Center, Baton Rouge, LA, United States

**Keywords:** brown adipocytes, fatty acid oxidation, *de novo* fatty acid synthesis, mitochondrial substrate utilization, uncoupled respiration

## Abstract

Brown adipocytes is a specialized fat cell that dissipates nutrient-derived chemical energy in the form of heat, instead of ATP synthesis. This unique feature provides a marked capacity for brown adipocyte mitochondria to oxidize substrates independent of ADP availability. Upon cold exposure, brown adipocytes preferentially oxidize free fatty acids (FFA) liberated from triacylglycerol (TAG) in lipid droplets to support thermogenesis. In addition, brown adipocytes take up large amounts of circulating glucose, concurrently increasing glycolysis and *de novo* FA synthesis from glucose. Given that FA oxidation and glucose-derived FA synthesis are two antagonistic mitochondrial processes in the same cell, it has long been questioned how brown adipocytes run FA oxidation and FA synthesis simultaneously. In this review, I summarize mechanisms regulating mitochondrial substrate selection and describe recent findings of two distinct populations of brown adipocyte mitochondria with different substrate preferences. I further discuss how these mechanisms may permit a concurrent increase in glycolysis, FA synthesis, and FA oxidation in brown adipocytes.

## Introduction

While white adipocytes primarily store excess energy in the form of triacylglycerol (TAG), brown adipocytes located in the interscapular brown adipose tissue (BAT) have a marked capacity to oxidize nutrients and dissipate energy as heat (1). Brown-like beige adipocytes also emerge within white adipose tissue (WAT) during prolonged cold exposure or pharmacological stimulation of β_3_-adrenergic receptors (2–5). Notably, activation of brown and beige adipocytes in rodents (6, 7) and humans (8–14) increases energy expenditure and improves systemic glucose and lipid homeostasis. Thus, brown/beige adipocytes have emerged as an appealing target against obesity and its related metabolic disorders, such as type 2 diabetes, insulin resistance, and dyslipidemia.

Upon activation, brown adipocytes simultaneously increase glycolysis, glucose-derived *de novo* fatty acid synthesis (FAS), and fatty acid oxidation (FAO) (15–19), which are mutually exclusive pathways in the same cell (20). In most mammalian cells, elevated glycolysis and subsequent pyruvate oxidation in the mitochondria block mitochondrial FAO. Conversely, elevated FAO decreases the activity of glycolytic enzymes in the cytosol and pyruvate dehydrogenase (PDH) within the mitochondrial matrix, thus leading to inhibition of pyruvate production, oxidation, and *de novo* FAS. It is not fully understood how brown adipocytes simultaneously increase glycolysis and FAS while primarily oxidizing FA in the mitochondria. To target brown adipocytes therapeutically, it is important to understand the underlying mechanism responsible for this unique phenomenon. This mini review will focus on recent advances in our understanding of substrate utilization in brown adipocytes and discuss molecular mechanisms that may permit the concurrence of glycolysis, FAS, and FAO in brown adipocytes.

## UCP1-mediated proton leak: A mechanism for high substrate oxidation in brown adipocytes

**Figure 1:**
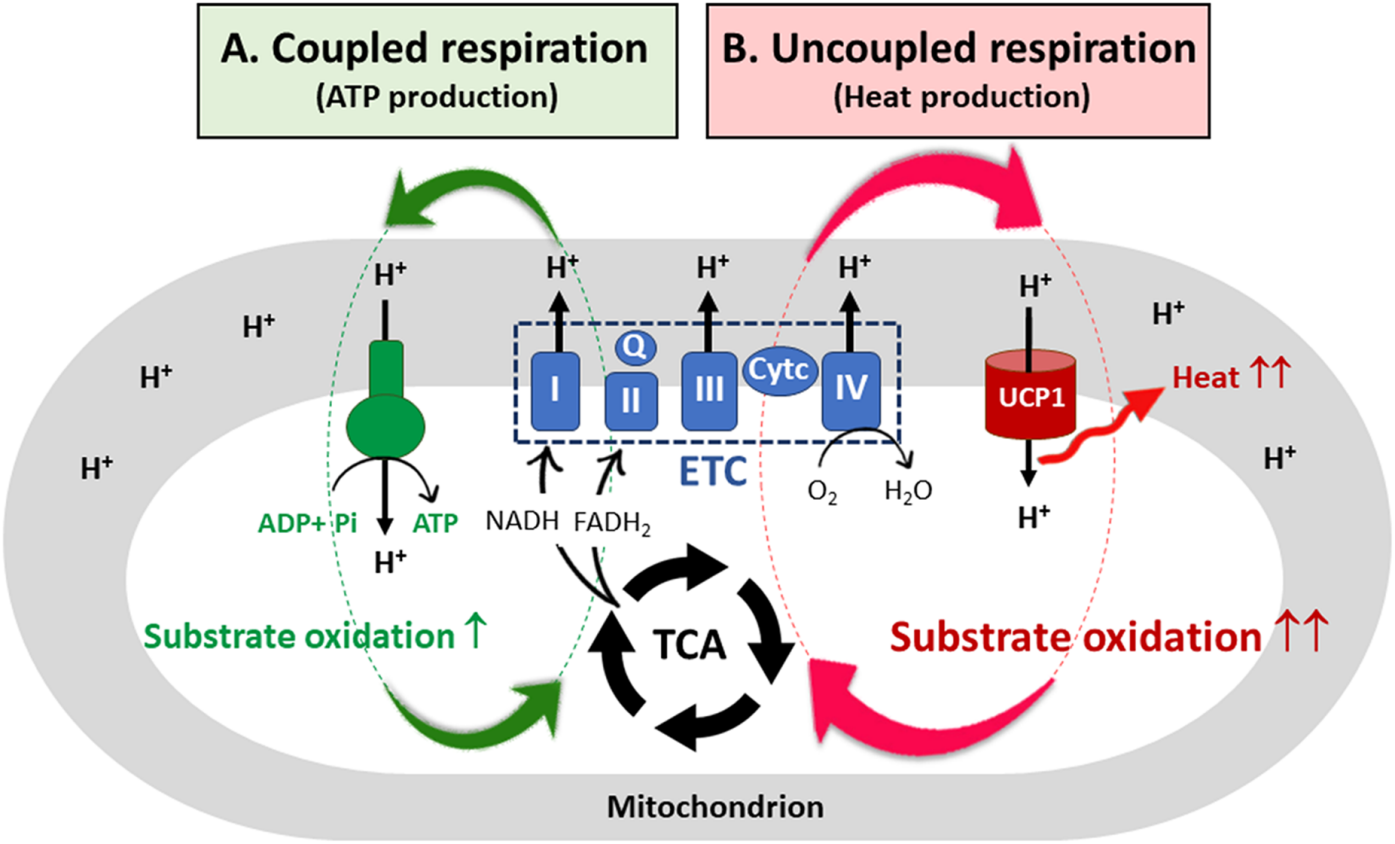
UCP1-mediated uncoupled respiration and its contribution to substrate oxidation. **(A)** In most mammalian cells, a proton motive force (PMF) created by the electron transfer chain (ETC) system is used by ATP synthase, resulting in ATP production (coupled respiration). To re-establish the electrochemical proton gradient across the inner mitochondrial membrane (IMM), the cells increase substrate oxidation, generating more NADH and FADH_2_ needed by the ETC. However, for coupled respiration, the rate of substrate oxidation and electron flow through the ETC is highly dependent on ADP availability. **(B)** In brown adipocytes, the protons pass from the intermembrane space into the mitochondrial matrix through the membrane bound UCP1. The resulting proton leak causes a drop in the PMF, releasing heat but not ATP synthesis (uncoupled respiration). The futile cycle of ETC-mediated proton pumps and UCP1-mediated proton leak provides a marked capacity for brown adipocyte mitochondria to oxidize substrates independent of ADP availability. TCA, the tricarboxylic acid cycle; I, II, III, IV, ETC multi-subunit complexes I through IV; Q, coenzyme Q; Cyt c, cytochrome c; UCP1, uncoupled protein 1.

In mammalian cells, oxidation pathways of glucose, FA, and amino acids converge onto a common pathway, the tricarboxylic acid (TCA) cycle, which generates NADH and FADH_2_ in the mitochondrial matrix. These reduced electron carriers donate their electrons to the electron transport chain (ETC) system, which is composed of four multi-subunit complexes (I-IV) located in the inner mitochondrial membrane (IMM) and two mobile electron carriers coenzyme Q and cytochrome c (21, 22). Subsequent electron transfer through the ETC leads to pumping of protons (H^+^ ions) from the mitochondrial matrix to the intermembrane space, creating an electrochemical proton gradient, also known as the proton motive force (PMF). The PMF is a form of potential energy composed of an electrical charge gradient (ΔΨ_m_) and a chemical gradient (ΔpH) across the IMM (23). The resulting PMF is used by F_0_F_1_-ATP synthase (**Figure 1A**; coupled respiration). The protons pass from the intermembrane space into the matrix through the F_0_ component, causing a conformational change in F_0_F_1_-ATP synthase so that ATP is produced from ADP and inorganic phosphate (24). For the coupling of ETC-mediated proton pumps to the ATP synthesis, the rate of substrate oxidation and electron flow is highly dependent on the availability of ADP (25). Fluctuations in coupling between ETC activity and ATP production can cause electron leak from the ETC onto oxygen, resulting in production of reactive oxygen species (ROS) (26).

Brown adipocytes contain a large number of mitochondria that uniquely express uncoupling protein 1 (UCP1) in the IMM (27, 28) along with abundant expression of TCA cycle enzymes and ETC complexes (I-IV) (1). Membrane-bound UCP1, an H^+^ transport protein, allows the re-entry of protons into the mitochondrial matrix independent of ATP synthesis (**Figure 1B**; uncoupled respiration) (29, 30). Thus, UCP1-mediated proton leak causes a drop in the PMF, and energy is lost as heat. As a mechanism to re-establish the electrochemical proton gradient (ΔpH) across the IMM, brown adipocytes increase the rate of substrate oxidation, generating more NADH and FADH_2_ needed by the ETC. Consequently, the futile cycle of ETC-mediated proton pumps and UCP1-mediated proton leak provides a marked capacity for brown adipocyte mitochondria to oxidize substrates without being affected by ADP availability. In addition, dissipation of the PMF by UCP1 has been shown to reduce mitochondrial ROS production, contributing to an increase in ETC complexes (31–33).

## Mitochondrial substrate utilization in brown adipocytes: Fatty acids vs glucose?

Brown adipocytes oxidize substantial amounts of substrates due to high ETC activity (proton pumping) and UCP1 activity (proton leak) in the IMM. Upon adrenergic stimulation of the cell by cold-dependent activation of the sympathetic nervous system or β_3_-adrenergic receptor agonists, brown adipocytes liberate free fatty acids (FFA) by lipolysis of TAG stored in lipid droplets as well as take up extracellular nonesterified fatty acids (NEFA) from the circulation (1, 34–38). FFA released from the intracellular TAG is the main source of FA for thermogenesis. Circulating NEFA is directed toward TAG replenishment in brown adipocytes although a portion of NEFA contributes to thermogenesis (35, 39). These FAs are ligated to CoA groups before being converted to acyl-carnitine for mitochondrial import through carnitine palmitoyltransferase 1 (CPT1) located in the outer mitochondrial membrane (OMM). Subsequent FA β-oxidation in the matrix produces acetyl-CoA, which then enters the TCA cycle as citrate after condensation with oxaloacetate (OAA). FAO results in a larger increase in acetyl-CoA levels per molecule of nutrient than glucose-derived pyruvate oxidation (i.e., 1 C_16_-palmitic acid generates 8 acetyl-CoA; 1 glucose generates 2 acetyl-CoA). Accordingly, FAO is more efficient in generating NADH and FADH_2_ and FA has been viewed as the primary substrate for energy-demanding brown adipocyte mitochondria (1, 6). Moreover, it is interesting to note that FA is not only the energy substrate for thermogenesis but also activates the UCP1-mediated proton leak across the IMM (29, 40).

Surprisingly, activated brown adipocytes also take up large amounts of glucose from the circulation while primarily utilizing FA to fuel thermogenesis (19, 41–45). However, recent studies have further found that the primary function of this glucose is not to support thermogenesis (<15%) but instead fuel *de novo* lipogenesis (DNL) through multiple mechanisms (16, 17, 19, 38, 42, 46–49): i) Pyruvate-derived citrate serves as the precursor for *de novo* FA synthesis (FAS); ii) Glucose-derived glycerol-3-phosphate serves as the structural backbone for TAG synthesis; iii) Glycolytic intermediates support the pentose phosphate pathway generating NADPH needed for DNL; and iv) Cytosolic ATP production during glycolysis meets energy requirement for DNL as well as compensates for the loss of mitochondrial ATP synthesis. Therefore, it has been suggested that, while oxidizing FA to fuel thermogenesis, brown adipocytes concurrently increase glycolysis and *de novo* FAS to replenish intracellular TAG pool in lipid droplets (15–19). However, it is currently unclear how brown adipocytes concurrently perform FAO and FAS in the same cell because these processes are two mutually exclusive pathways in healthy cells.

More interestingly, recent studies have shown that cold-activated BAT in rodents and humans utilizes additional substrates such as branched-chain amino acids (BCAA) (50, 51), glutamate (44), and succinate (52) to support thermogenesis. Extracellular succinate contributes to thermogenic respiration in BAT by the succinate dehydrogenase (SDH)-mediated oxidation in the TCA cycle (52), although its relative contribution to thermogenesis is unclear. Similarly, BCAAs and glutamate enter the TCA cycle as acetyl-CoA/succinyl-CoA and α-ketoglutarate, respectively, to generate more reducing equivalents in BAT (44, 50, 51). It is also possible that carbons from these additional substrates replenish TCA cycle intermediates that leave the cycle for biosynthetic pathways (e.g., citrate for *de novo* FA synthesis).

## Molecular mechanisms regulating mitochondrial substrate selection

In most mammalian cells, mitochondrial FAO suppresses glycolysis, pyruvate oxidation, and *de novo* FAS (20). FAO-induced increases in acetyl-CoA/CoA, NADH/NAD^+^, and ATP/ADP ratios inhibit the activity of pyruvate dehydrogenase (PDH) that catalyzes the conversion of glucose-derived pyruvate to acetyl-CoA in the mitochondria (53, 54). The resulting decrease in acetyl-CoA reduces the production of pyruvate-derived citrate that exits the mitochondria to serve as the precursor for FAS. Thus, FAO-dependent inhibition of PDH activity in the mitochondria is the primary mechanism preventing both pyruvate oxidation and *de novo* FAS from glucose. Additionally, FAO can inhibit glycolysis. A portion of excess citrate produced from FA-derived acetyl-CoA is exported to the cytosol, where it in turn inhibits glycolytic enzymes, such as phosphofructokinases (PFK1, PFK2) and pyruvate kinase (PK) (20, 55–57).

Conversely, when extracellular glucose increases, enhanced glycolysis provides more pyruvate to the mitochondria. The conversion of pyruvate to acetyl-CoA by PDH and to OAA by pyruvate carboxylase (PC) increases citrate production in the mitochondria. Under high glucose, excess citrate is exported to the cytosol and hydrolyzed back to acetyl-CoA and OAA by ATP-citrate lyase (ACLY). Acetyl-CoA is then carboxylated to malonyl-CoA by two acetyl-CoA carboxylases, ACC1 and ACC2 (58). Malonyl-CoA is the precursor of *de novo* synthesized FA. Remarkably, malonyl-CoA produced by ACC2 allosterically inhibits CPT1 (59, 60) that controls the entry of long-chain fatty acids from the cytosol into mitochondria. By this mechanism, glucose-derived malonyl-CoA prevents the oxidation of newly synthesized and pre-existing FAs. Thus, malonyl-CoA is a key metabolite regulating the balance between FAS and FAO.

Although ACC1 and ACC2 have same enzyme activity with over 70% protein sequence similarity, they play distinct roles in the control of FAS and FAO (58, 61, 62). ACC1 is cytosolic and directs malonyl-CoA toward *de novo* FA synthesis catalyzed by fatty acid synthase (FAS). In contrast, ACC2 is associated with the OMM and regulates FAO through malonyl-CoA-mediated CPT1 inhibition (59–61, 63). While lipogenic WAT predominantly expresses ACC1, BAT expresses similar amounts of ACC1 and ACC2 (64). In addition, BAT expresses CPT1β, an isoform with high sensitivity to malonyl-CoA (65–67). Despite the expression of ACC2 and CPT1β, BAT mitochondria have the highest CPT1 activity among the tissues expressing CPT1β (65–67). High FAO in BAT is surprising in light of the inhibitory effect of malonyl-CoA produced by ACC2 on CPT1β-mediated FA transport. It is unclear whether ACC2 activity or association to the mitochondria is negatively regulated by cold in brown adipocytes.

It is interesting to note that concurrent FAO and FAS have also been observed in a subset of cancer cells (68–70). Glycolytic colorectal cancer cells recruit FAO as an adaptive response to extracellular acidification associated with increased pyruvate to lactate conversion (68). A selective decrease in the transcription of *ACACB* gene under acidosis was in part the mechanism permitting mitochondrial FAO. However, it is unlikely that the selective decrease in *ACACB* gene expression provides a mechanism by which brown adipocytes concurrently perform FAO and FAS because BAT upregulates the expression of both *ACACA* and *ACACB* genes encoding ACC1 and ACC2, respectively, in response to cold (17). As another example, a subset of highly proliferating B-cell lymphomas concurrently stimulates mitochondrial FAO while increasing glycolysis and FAS (69); however, the underlying mechanism remains unknown.

## Heterogeneity of brown adipocytes in BAT

Single-cell and single-nucleus RNA sequencing of BAT has uncovered the existence of multiple brown adipocyte subpopulations with large variability in their transcriptomes and with different degrees of thermogenic capacities (71–73). Compared with the high-thermogenic brown adipocytes, low-thermogenic brown adipocytes express lower levels of Ucp1 along with reduced mitochondrial respiration (71). It is considered that these subpopulations are derived from distinct precursor cells and/or represent different cell states acquired during environmental temperature changes (71–73). The co-existence of functionally different brown adipocytes within the BAT may in part explain how BAT performs FAO and FAS simultaneously. Further studies are required to delineate the location, functional specialization, and substrate utilization of these brown adipocyte subpopulations and their ratios in response to environmental stimuli.

## Heterogeneity of mitochondria within the brown adipocyte: FA-oxidizing vs lipogenic mitochondria

**Figure 2:**
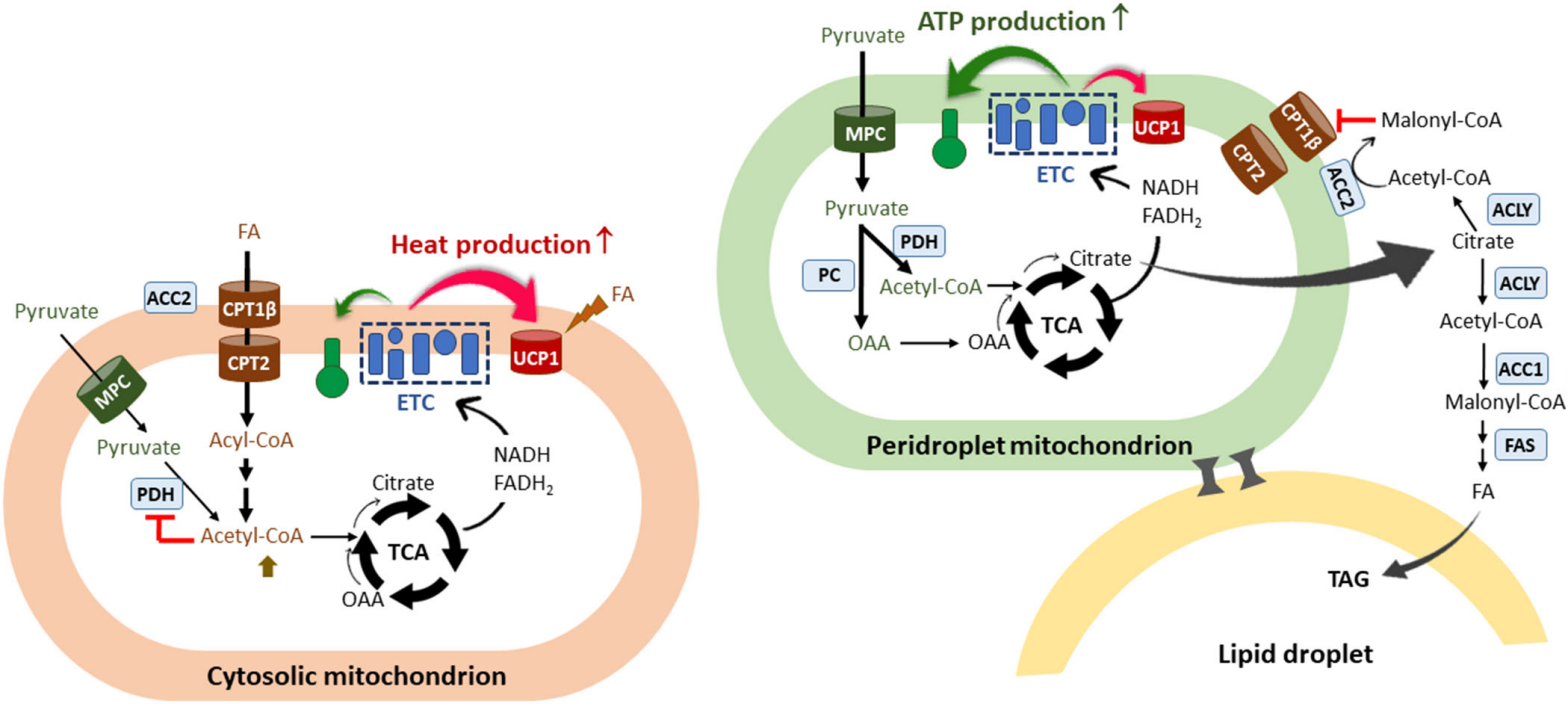
The co-existence of two functionally different mitochondria within the brown adipocyte. A scheme of two types of mitochondria identified in the brown adipocyte: cytosolic mitochondria (CM) and peridroplet mitochondria (PDM) (74–78). PDM are anchored to the lipid droplets and segregated from the pool of CM. CM preferentially oxidize FA and are more thermogenic compared to PDM. FAO-induced increases in acetyl-CoA/CoA and NADH/NAD^+^ ratios would inhibit PDH in the matrix, resulting in a decrease in pyruvate-derived citrate production and subsequent malonyl-CoA accumulation in close vicinity of CM. FA-derived citrate would be rapidly oxidized through the TCA cycle to support UCP1-mediated thermogenesis. On the contrary, PDM have a higher capacity for pyruvate oxidation and ATP synthesis compared to CM. Given that coupled respiration is dependent on ADP availability, excess citrate would escape from the mitochondria and be converted to malonyl-CoA by ACC1 and ACC2, thus contributing to *de novo* lipogenesis and simultaneously preventing CPT1β-mediated FA entry into PDM. The co-existence of two functionally different mitochondria within the brown adipocyte may in part explain the concurrence of glycolysis, FA synthesis, and FA oxidation in brown adipocytes. FA, fatty acids; ETC, electron transport chain; CPT, carnitine palmitoyltransferase; UCP1, uncoupled protein 1; TCA, the tricarboxylic acid cycle; OAA, oxaloacetate; MPC, mitochondrial pyruvate carrier; PDH, pyruvate dehydrogenase; PC, pyruvate carboxylase; ACLY, ATP-citrate lyase; ACC, acetyl-CoA carboxylases; FAS, fatty acid synthase; TAG, triacylglycerol.

In addition to heterogeneity of brown adipocytes, recent studies have demonstrated the presence of metabolically distinct populations of mitochondria within the same brown adipocyte: cytosolic mitochondria (CM) and peridroplet mitochondria (PDM) (74–78). PDM are found to be anchored to the lipid droplets and have reduced motility and fusion-fission dynamics that segregate PDM from the rest of the mitochondrial population (79, 80). While CM preferentially oxidize FA for theromgenesis, PDM have a higher capacity for pyruvate oxidation and ATP synthesis (74) (**Figure 2**). In line with increased oxidative phosphorylation, PDM is enriched with ATP synthase compared to CM (74) although UCP1 levels are comparable in PDM and CM (74, 78). More interestingly, an increase in PDM is associated with lipid droplet expansion in brown adipocytes (74). Given that coupled respiration is dependent on ADP availability, excess citrate produced from pyruvate-derived acetyl-CoA in the PDM may exit the mitochondria and be converted to malonyl-CoA by ACC1 and ACC2, thus contributing to *de novo* FAS for TAG synthesis and concurrently preventing FA entry into these specific subpopulations of mitochondria (**Figure 2**). On the contrary, in CM preferentially oxidizing FA (74), FA-derived acetyl-CoA could inhibit PDH activity, resulting in a decrease in pyruvate-derived citrate production and subsequent malonyl-CoA accumulation in close vicinity of CM (**Figure 2**). It is unclear whether there is a difference in ACC2 levels between PDM and CM. CM could maximize UCP1-mediated thermogenesis by producing high levels of NADH and FADH_2_ from FAO. The resulting rapid oxidation of FA-derived citrate through the TCA cycle may prevent citrate export to the cytosol for inhibition of glycolytic enzymes.

The association between mitochondria and lipid droplets has been overserved in other tissue/cell types including skeletal muscle, heart, and adipocytes (77, 81–83). In contrast to the lipogenic role of PDM in brown adipocytes, several studies reported conflicting results that PDM promotes the oxidation of FA released from lipid droplets (77, 81). This discrepancy may imply that the role of PDM is differently regulated by the cell type, nutritional status, or cellular stress. Proteome profiling of PDM and CM in BAT has identified a subset of mitochondrial proteins differentially expressed between PDM and CM although their impact on the functional difference has not been explored (78). Additional studies are required to quantitatively characterize PDM and CM mitochondrial proteins (e.g., MPC1/2, ACC2, CPT1β) and understand the significance of relative PDM/CM ratio and the mechanism controlling this ratio in brown adipocytes.

## Conclusion

Brown adipocytes have two unique features: (1) UCP1-mediated dissipation of the PMF, which provides a mechanism for maximal substrate oxidation in the mitochondria and (2) concurrence of glycolysis, *de novo* FAS, and FAO. Upon activation, brown adipocytes increase glycolysis and *de novo* FAS to replenish intracellular TAG pools that are depleted due to increased lipolysis and FAO. The co-existence of FA-oxidizing and lipogenic mitochondria within the brown adipocyte in addition to heterogeneity of brown adipocytes may in part explain the unique capacity of brown adipocytes to be involved simultaneously in FAO and FAS.

## Author contributions

The author confirms being the sole contributor of this work and has approved it for publication.
